# Undernutrition in Patients with COPD and Its Treatment 

**DOI:** 10.3390/nu5041316

**Published:** 2013-04-18

**Authors:** Masayuki Itoh, Takao Tsuji, Kenji Nemoto, Hiroyuki Nakamura, Kazutetsu Aoshiba

**Affiliations:** Department of Respiratory Medicine, Tokyo Medical University Ibaraki Medical Center, 3-20-1 Chuou, Ami, Inashiki, Ibaraki, 300-0395, Japan; E-Mails: fraser@tokyo-med.ac.jp (M.I.); tak1924@tokyo-med.ac.jp (T.T.); nemoto@tokyo-ed.ac.jp (K.N.); hiroyuki@tokyo-med.ac.jp (H.N.)

**Keywords:** COPD, undernutrition, adipokines, hormones, nutritional supplement therapy, vitamins

## Abstract

Chronic obstructive pulmonary disease (COPD) is a chronic inflammatory disorder of the lung and whole body caused mainly by tobacco smoking. Patients with advanced COPD are in a state of undernutrition, referred to as pulmonary cachexia; the exercise performance and quality of life (QOL) of these patients are deteriorated, the vital prognosis is unfavorable, and the medico-economic burden posed by poorly nourished COPD patients is high. The mainstays of COPD treatment are pharmacotherapy, mainly with bronchodilators, and non-pharmacotherapeutic approaches such as respiratory rehabilitation and nutrition counseling. Nutritional supplement therapy, consisting primarily of high calorie intake, has been demonstrated to be effective for maintaining and improving the muscle strength and exercise tolerance in poorly nourished COPD patients. The efficacy of intake of various nutrients, besides a high calorie intake, for amelioration of the disease state of COPD has also been reported. The roles of adipokines in the pathophysiology of COPD have begun to receive attention recently, and not only their regulatory effects on appetite and nutritional status, but also their influence on systemic inflammation have been increasingly clarified. We review the papers on COPD and nutrition and discuss the role of nutritional supplement therapy in the treatment of COPD.

## 1. Introduction

Chronic obstructive pulmonary disease (COPD) is a chronic inflammatory disorder of the lung and whole body caused mainly by tobacco smoking, and is characterized by progressive, persistent airflow obstruction. There has been a global trend of increase in the number of patients with COPD, and as estimated by the World Health Organization (WHO), COPD will become the third leading cause of death in the world in 2020. Such a progressive increase in the number of patients with COPD, especially of patients with severe disease, will pose a considerable medico-economic burden; therefore, preventive measures and therapies for the disorder are being assiduously pursued.

Most patients with severe COPD are lean, and not infrequently in a state of marked undernutrition, referred to as pulmonary cachexia. In fact, 25% to 40% of COPD patients are in an undernourished state [1,2], and low body weight and low fat-free mass (FFM) have been recognized as unfavorable prognostic factors in patients with COPD [3,4,5]. Nutritional supplement therapy has long been prescribed for the management of such lean patients with COPD. While past study reports had not borne out testimony to the efficacy of nutritional supplement therapy, a recent meta-analysis [6] has not only verified the efficacy of nutritional supplement therapy in lean patients with COPD, but also demonstrated that the effect of nutritional supplement therapy can be augmented by concomitant pulmonary rehabilitation. This manuscript briefly reviews the recent papers dealing with COPD and nutrition, and discusses the role of nutritional supplement therapy in the management of COPD.

## 2. State of Undernutrition in COPD Patients

Patients with advanced COPD often show decreased body weight. Weight loss is noted in 25%–40% of all COPD patients, with 25% of patients with moderate to severe disease and 35% of patients with extremely severe disease showing a reduced free fat mass (FFM) index [1,2]. Decreased body weight has been identified as a poor-prognostic factor in patients with COPD [3,4,5], and the survival time is reported to be only 2 to 4 years in patients with severe disease who are lean and have a forced expiratory volume % in one second (FEV_1_%) of less than 50%. It has also been reported that COPD patients with a body mass index (BMI) of <20 kg/m^2^ have a higher risk of acute exacerbations as compared to COPD patients with a BMI of 20 kg/m^2^ or greater, and that patients exhibiting weight loss during a 1-year observation period are more prone to acute exacerbations than those who do not exhibit weight loss over the same period [7]. A study that analyzed the data of COPD patients hospitalized with acute exacerbations demonstrated a positive correlation of the body weight with the FEV_1_% and a negative correlation between the BMI and duration of hospitalization [8]. Furthermore, body weight has been demonstrated to be positively correlated with the forced expiratory volume in one second (FEV_1_), exercise tolerance and diffusing capacity of the lung, even in patients with stable-phase COPD [3]. The rate of post-exercise decrease in the concentration of reduced glutathione (GSH) in the vastus lateralis muscle has been reported to be higher in COPD patients with a low BMI [9]. This might suggest that for a given level of exercise intensity, patients with advanced emaciation and those with diminished muscle mass may be more vulnerable to the impact of oxidative stress. Thus, low body weight and undernutrition are factors that adversely affect the prognosis and locomotor capacity in COPD patients.

## 3. Causes of Undernutrition in COPD Patients

As causes of undernutrition in COPD patients, energy insufficiency due to decreased dietary intake caused by appetite loss associated with diminished general physical activity, a depressive tendency, or dyspnea while eating can be speculated [10]. Secondly, enhanced energy expenditure due to increased work of breathing may also account for the undernutrition. The resting energy expenditure (REE) is increased in COPD patients [11] and increased REE has been demonstrated in lean COPD patients [12]. As the third major cause of undernutrition in COPD patients, effects of humoral factors such as inflammatory cytokines, adipokines, and hormones on nutrition have been pointed out. COPD is generally recognized as a systemic inflammatory disorder not involving the lungs alone, and is characterized by increased production of inflammatory cytokines such as interleukin (IL)-6, IL-8, and tumor necrosis factor (TNF)-α, and also of chemokines [13,14]. Marked increase of TNF-α production from the peripheral blood monocytes has been demonstrated in extremely lean COPD patients [15]. A significant correlation has also been shown between IL-6 production and decreased appetite [16]. However, obese subjects are known to have elevated serum IL-6 levels [17], probably as a result of enhanced IL-6 production from white adipose tissue [18]. A recent study showed that COPD patients had elevated serum IL-6 levels, with no significant difference in the level between lean and obese patients [19]. Thus, the role of IL-6 in undernutrition and overnutrition in COPD patients remains unclear.

## 4. Adipokines and Hormones Affecting the Nutritional Status in COPD

Adipokines is a generic term for bioactive proteins secreted by adipocytes, including leptin, adiponectin, TNF-α and IL-6, and adipokines have been shown to play important roles in regulating the appetite and influencing the nutritional status [20]. In recent years, it has been demonstrated that dysregulation of adipokines gives rise to low-grade systemic inflammation in COPD [21]. Reports on the relationship of various adipokines with the pathophysiologic state of COPD are summarized in Table 1. 

The adipokine leptin was discovered as a factor regulating appetite and energy expenditure, which is promptly secreted in response to food ingestion to suppress appetite and enhance energy expenditure. Experiments *in vitro* have shown that inflammatory cytokines such as IL-1 and TNF-α induce leptin secretion [22]. An observational study has shown that COPD patients with BMI ≤ 21 (*n* = 14) had lower blood levels of leptin than normal weighted COPD patients (*n* = 16) and healthy control subjects (*n* = 20) [23]. Other studies have also shown that the blood levels of leptin and TNF-α were increased during acute exacerbations in COPD patients (*n* = 52) [24] while there was no significant relationship between the blood leptin levels and blood TNF-α levels in stable COPD patients (*n* = 31) [25]. It has also been reported that leptin possesses a proinflammatory effect, so that a deficiency of leptin in mice leads to an increased susceptibility to infections [26]. In this context, increased leptin expression in bronchial epithelial cells obtained from COPD patients (*n* = 27) has been reported with increasing disease severity and to be correlated with the inhibition of inflammatory cell apoptosis [27]. Leptin is also produced by type II alveolar epithelial cells and alveolar macrophages, and the possibility of regulation by leptin of the immune responses to cope with inhaled hazardous substances has also been pointed out [28]. In moderate COPD patients, sputum leptin levels were correlated with the sputum TNF-α levels but were inversely correlated with the plasma leptin levels, suggesting that measurements of the sputum leptin may be useful for assessing the severity of local lung inflammation [29].

**Table 1:** Observed concentrations of the blood levels of inflammatory cytokines, adipokines and hormones in patients with chronic obstructive pulmonary disease (COPD).

Cytokines, Adipokines and Hormones	Primary Source	Functions	Changes in Blood Levels in COPD Patients	References
Adiponectin	Adipocytes	Insulin sensitizer, suppress inflammation	↑ compared to healthy smokers and nonsmkers; correlated with the residual volume, blood TNF-α levels and mortality; inversely correlated with the predicted %FEV1; ↑ during acute exacerbations	[30,31,32,33,34]
Leptin	Adipocytes, bronchial epithelial cells, alveolar type II pneumocytes, lung macrophages	Appetite control, promote inflammation; regulate hematopoiesis, angiogenesis, wound healing	↓ in COPD patients with low BMI compared to COPD patients with normal and high BMI and healthy control; ↑ and correlated with TNF-α during acute exacerbation; not correlated with TNF-α in stable COPD patients; plasma and sputum leptin levels are inversely correlated	[23,24,25,29]
Resistin	Peripheral blood mononuclear cells, adipocytes	Promote insulin resistance and inflammation through IL-6 and TNF-α production	Inversely correlated with FEV_1_% predicted	[35]
TNF-α	Stromal vascular fraction cells, adipocytes, monocytes	Promote inflammation; antagonize insulin signaling	↑ compared to healthy control; ↑ production from the peripheral blood monocytes in lean COPD patients	[13,14,15]
IL-6	Adipocytes, atromal vascular fraction cells, liver, muscle	Promote inflammation; appetite loss	↑ compared to healthy control	[14]
Ghrelin	X/A-like cells	Stimulate appetite and GH release	↑ in underweight patients compared with normal weight patients and healthy control subjects; positively correlated with residual lung volume; inversely correlated with FEV_1_% predicted	[36]

Abbreviations: ↑, increase; ↓, decrease.

Many studies have also been published concerning adiponectin and the disease state of COPD. Adiponectin is a protein secreted from adipocytes, just like leptin, but with opposite actions: adiponectin enhances appetite and acts to reduce the fatty acids in muscle tissues. Furthermore, adiponectin also has anti-inflammatory, anti-diabetic and anti-arteriosclerotic effects [30], and is hence known as a beneficial adipokine. The blood adiponectin level is elevated in COPD patients, with the levels reported to be positively correlated with elevated plasma TNF-α levels as well as with an increase in the residual lung volume, and to be negatively correlated with the body weight [31]. Elevation of the blood adiponectin levels in COPD patients (*n* = 71) has also been shown to be related to a decline in the ratio of the actually measured forced expiratory volume in one second (FEV_1_) to the predicted FEV_1_ (FEV_1_% predicted) [32]. There is a proven positive correlation between the mortality rate and elevated blood adiponectin levels in patients with COPD [33]. A prospective cohort study with 170 stable COPD patients has shown that the blood adiponectin level increases during acute exacerbations of COPD, representing a marker of systemic inflammation in COPD exacerbations [34]. Thus, adiponectin production appears to be upregulated in COPD patients as a protective mechanism that counteracts the effects of pro-inflammatory cytokines.

Contrary to the case in severe COPD patients, in patients with mild-to-moderate COPD, the primary cause of death is ischemic cardiovascular disease, for which obesity is an important risk factor [37]. The Third National health and Nutrition Examination Survey (NHANESIII) revealed that extreme obesity (BMI > 40) was associated with a significantly increased risk of death in both patients with respiratory disease and chronic lower respiratory disease [38]. Enhanced fat accumulation, especially in the visceral compartment, in obese COPD patients is associated with all-cause and cardiovascular disease mortality, probably as a result of increased production of IL-6 from visceral fat [39]. Given that serum adiponectin levels are decreased in obese subjects [40], decreased adiponectin levels in obese COPD patients may also contribute to an increased risk of death through exerting proinflammatory effects and enhancing the cardiometabolic risk.

Resistin, another adipokine, has been shown to have an insulin resistance-inducing effect and to induce inflammation via stimulation of TNF-α and IL-6 production by monocytes [35]. However, there have been few reports regarding the biodynamic behaviors of resistin in COPD.

Changes in the blood levels of adipokines are known to differ between the two genders. For example, it has been reported that while the blood levels of leptin, adiponectin and resistin in male patients with COPD have not been shown to differ from those in healthy male individuals, in female COPD patients, especially obese women with COPD, the blood leptin levels are known to be elevated. Gender differences in leptin secretion and the influences of the sex hormones are assumed to account for this phenomenon [19].

Ghrelin is also known as a hormone involved in regulating the nutritional status. It is a peptide hormone that was first extracted from the gastric wall [41] and, besides its appetite-stimulating effect [42], it also stimulates growth hormone (GH) secretion to increase the fat and muscle mass [41]. Thus, ghrelin is considered to have antagonistic effects to those of leptin.

Plasma ghrelin concentrations have been reported to be significantly higher in lean COPD patients (*n* = 26) than in patients of normal body weight (*n* = 24) and healthy controls (*n* = 13). Plasma ghrelin concentrations have been shown to be inversely correlated with the BMI and positively correlated with the lung residual volume, and plasma ghrelin concentrations have been reported to increase with progression of the severity of COPD [36]. It is considered probable that ghrelin secretion increases in lean COPD patients to rectify the low body weight. In a multicenter, randomized controlled trial (RCT) study carried out in lean COPD patients (*n* = 33), intravenous injections of ghrelin (2 μg/kg, twice a day) for three weeks resulted in an increase in the food intake, with consequent recovery of the body weight and FFM, along with strengthening of the respiratory muscles [43]. Furthermore, it was reported that respiratory rehabilitation combined with intravenous ghrelin therapy for three weeks resulted in improvement of the respiratory muscle strength and improvement of the St. George’s Respiratory Questionnaire (SGRQ) score, used to evaluate the health-related quality of life (HRQoL), by Week 7 after the start of treatment [44].

The use of recombinant human growth hormone (rhGH) has been studied extensively in various critically ill patients [45]. A RCT in which rhGH (0.15 IU/kg) was administered for three weeks during a two-month course of respiratory rehabilitation in COPD patients revealed an increase in the lean body mass in the rhGH-treated group (2.3 ± 1.6 kg) and in the control group (1.1 ± 0.9 kg) (*p* < 0.05), with no changes observed in respect of the exercise tolerance (6-min walking distance, 6 MWD), respiratory muscle strength, or the grip strength [46].

## 5. Nutritional Supplement Therapy for COPD Patients

In regard to nutritional supplement therapy in patients with COPD, the results of a meta-analysis in the Cochrane Review published in 2005 indicated a low likelihood of efficacy of the therapy in terms of improvement of the body weight, muscle mass and respiratory function [47]. Nevertheless, subsequent reports demonstrated the efficacy of this therapy [48]; hence, until recently, there was no unanimity of opinion about the efficacy of nutritional supplement therapy in patients with COPD. However, a meta-analysis published in 2012 revealed weight gain and improvement of the grip strength in COPD patients receiving nutritional supplement therapy [6]. In the Cochrane Review updated in 2012 also [49], results of a meta-analysis of data from 17 RCTs revealed that nutritional supplement therapy produced body weight recovery and raised the FFM index, with consequent improvement of the exercise tolerance (6 MWD) in COPD patients who were undernourished. The results also provided evidence for a certain ameliorative effect of the therapy on the respiratory muscle strength (inspiratory and expiratory muscle strength) and HRQoL (SGRQ).

## 6. High-Calorie Nutrition Therapy for COPD

It is common practice for high-calorie nutrition therapy using dietary supplement beverages to be undertaken as nutritional supplement therapy in COPD patients (Table 2). High-fat, low-carbohydrate diets are recommended for lean COPD patients [50], however, it is not necessarily true that the greater the caloric intake, the more beneficial it is for the patients. In a study on COPD patients treated with a high-calorie dietary supplement solution at 125 mL/day (2380 kJ = 6.35 kJ/mL; 20% energy from protein, 60% from carbohydrate and 20% from fat) or 200 mL/day (3350 kJ = 4.19 kJ/mL; 22% energy from protein, 60% from carbohydrate and 18% from fat) for 8 weeks, the 125 mL dose group exhibited greater body weight gain than the 200 mL dose group [51]. Furthermore, in another study, two groups of patients ingested a dietary supplement solution containing whey protein, carbohydrates (1.5 kcal/mL; 20% energy from protein and 60% from carbohydrate) and antioxidants at a resting energy expenditure (REE) × 1.7 (Group A) or REE × 1.3 (Group B) in terms of the caloric intake, and were compared at Week 12 of the supplement therapy. Weight gain with increased body fat mass and worsened airflow limitation were more frequent among the patients of Group A, while increased body weight and muscle strength (grip strength) with improvement of airflow limitation and QOL were more frequent among the patients of Group B [52]. It might follow, therefore, that prescription of an appropriate caloric intake taking into consideration the nutrient composition, including the protein, carbohydrate and fat composition, of the diet are of greater importance in the nutritional supplement therapy of COPD patients than mere increase of the caloric intake. It is also important to consider supplementation of other specific nutrients as described later. Appropriate physical exercise is also effective in addition to an effort to increase the caloric intake. In a study with combined regimens consisting of nutritional supplement therapy and concomitant low-intensity respiratory rehabilitation over a period of 12 weeks, for example, increased body weight and FFM, improvement of the QOL and exercise tolerance, and reduced blood levels of inflammatory cytokines were noted [53].

**Table 2:** Results of randomized controlled trials (RCTs) of nutritional supplement therapy for COPD.

Nutrients	Effect on FEV_1_	Other Effects	Results of Meta-Analysis	References
Liquid prepared supplement	No change	Body weight, FFMI, 6MWD ↑; SGRQ improved	Positive	[6,49]
Fruits and vegetable	No change (12 weeks) Improve (3 years)	IL-8, TNF-α, CRP →	Not done	[54,55]
Vitamin E	No data	Risk of COPD ↓	Not done	[56]
Vitamin D	No change (1 year)	QOL, mortality rate, acute exacerbation rate →	Not done	[57]
Creatine	No data	6MWD, shuttle walk test, SGRQ, upper and lower limb strength →	Negative	[58,59,60,61]
Glutamine	No data	Lactate threshold, VO2 peak →; IL-6, IL-8, TNF-α → in exacerbated patients receiving mechanical ventilation	Not done	[62,63]
l-carnitine	No data	6MWD, inspiratory muscle strength ↑; blood lactate concentration after exercise ↓	Not done	[64]

Abbreviations: FFMI, free fat mass index; SGRQ, St. George Respiratory Questionnaire; 6MWD, 6-min walk distance; →, no change; ↑, increase; ↓, decrease.

## 7. Nutritional Supplement Therapy Other Than High-Calorie Nutrition Therapy: Intake of Fruits and Vegetables

Many cross-sectional cohort studies have demonstrated a correlation between the daily fruit and vegetable (FV) intake and the FEV_1_ in patients with COPD [65,66,67,68]. The results of a cross-sectional cohort study conducted over a period of 5–7 years revealed that low fruit intake was associated with a decrease of the FEV_1_ [69]. The results of another cross-sectional cohort study showed that the risk of development of COPD decreased with increasing fruit consumption, with the risk ratio of a ≥70 g/day intake group *vs.* a <14 g/day intake group being 0.68 [70]. A cross-sectional cohort study, which demonstrated that the risk of development of COPD decreased by 24% in subjects whose fruit intake was 100 g/day, has also been published [71]. Based on these study results, RCTs have been conducted to assess the efficacy of FV intake in COPD patients. In a RCT carried out by Keranis and colleagues [54], which was published in 2010, 120 patients with COPD were divided into a high FV intake group and a control group, and followed up by observation for three years. The observation revealed an improvement of the FEV_1_ in the high FV intake group as compared to that in the control group. In a RCT by Baldrick *et al.* [55] published in 2012, 81 COPD patients with a habitually low FV intake (two or fewer portions of FV per day) were randomized to a control group or the intervention group (five or more portions of FV per day) for 12 weeks. The observed increases in the serum zeaxanthin and β-cryptoxanthin levels confirmed that the intervention group ingested approximately 2- to 6-fold higher amounts of FV as compared to the control group. However, there were no significant differences in the respiratory function test data between the two groups. There were also no significant differences in the levels of inflammatory markers such as serum IL-8, TNF-α and CRP, or in the urinary or sputum levels of the oxidative stress marker 8-isoprostane. These negative results, nevertheless, do not totally negate the possible therapeutic efficacy of FV intake in COPD patients, but likely suggest that a longer duration of intervention might be needed to achieve an overt therapeutic effect of FV intake. To sum up the above studies, although it has been pointed out that FV intake may possibly prevent development of COPD, few clinical studies verifying the efficacy of FV intake have been published yet.

## 8. Intake of Vitamins

Effects on COPD of individual nutrients in foodstuffs rather than of the foodstuffs themselves have also been investigated. For example, study results demonstrating a diminished risk of development of COPD in subjects with an increased intake of dietary fibers contained in FV and grains have been presented [72]. The nutrients most extensively investigated in respect of their effects in COPD patients are vitamins, and many study reports have dealt particularly with vitamins C and E, both of which have antioxidant effects.

Vitamin C (ascorbic acid) is a water-soluble vitamin that is among the most important of dietary antioxidants, with a low toxicity, in mammals; it occurs in the cytoplasm and circulating plasma, and scavenges aqueous-phase radicals. Vitamin E, another antioxidant vitamin, on the other hand, is fat-soluble, occurring on the cell membranes and plasma lipoproteins. Several studies have examined the effects of exogenous vitamin C or E supplementation on oxidative stress in COPD patients. In a study carried out in COPD patients (*n* = 35) administered placebo, 400 mg/day vitamin E, 200 mg/day vitamin E or 250 mg/day vitamin C for 12 weeks, the results showed that vitamin E or C supplementation did not change the level of endogenous DNA damage of the white blood cells (WBC), but significantly suppressed H_2_O_2_-induced DNA breakage [73]. Another study in COPD patients (*n* = 30) treated with 400 IU vitamin E for 12 weeks demonstrated significant reductions in the plasma malondialdehyde (MDA) levels, although neither the plasma alpha-tocopherol levels and red blood cell superoxide dismutase levels, nor the lung function showed any significant changes after the vitamin E supplementation [74]. In regard to the relationship of the intakes of vitamins C and E to the pulmonary function, most cross-sectional studies in healthy subjects and COPD patients reported so far have shown that the FEV_1_ is well maintained in individuals with higher intakes of vitamin C and/or vitamin E [75,76,77,78,79,80,81,82]. Further, a large-scale RCT (Women’s Health Study [WHS], *n* = 38,597) demonstrated that the risk of development of chronic lung diseases, such as COPD, during a 10-year observation period was reduced by 10% in a group of subjects taking vitamin E (600 IU) on alternate days [56]. Meanwhile, the interactions between vitamins and antioxidant enzymes were also investigated. For example, it was reported that deficient vitamin C intake resulted in a marked depression of FEV_1_ in heavy tobacco smokers bearing a mutant gene encoding GCLC, a subunit of glutamate-cysteine ligase (GCL) involved in glutathione synthesis [83]. β-Carotene, contained in abundance in FV, is also endowed with a free radical-scavenging effect. A study report documents that the rate of aging-associated decrease of the FEV_1_ was greater in humans bearing a mutant gene for heme oxygenase-1 (HO-1), an antioxidant enzyme, and that the rate of aging-associated decrease of the FEV_1_ was delayed in subjects with a high intake of β-carotene [84]. Lycopene, which tomatoes and carrots contain in abundance, also has an antioxidant effect, and a positive correlation between lycopene intake and FEV_1_ has been reported in patients with COPD and bronchial asthma [85].

Vitamin D, contained in rich abundance in fishes such as swordfish, salmon and mackerel pike, and also in mushrooms, is a nutrient that regulates calcium homeostasis and bone metabolism in the body. Vitamin D deficiency has been noted in more than 60% of patients with severe COPD [86] and is thought to be a risk factor for concurrent osteoporosis [87]. Since vitamin D is known to have effects on immune reactions, inflammation, remodeling of airways and muscle strength [88,89,90,91,92,93], involvement of this vitamin in the pathophysiology of COPD is currently being investigated from various viewpoints. A cross-sectional cohort study in 14,091 ordinary persons based on the NHANES III reported in 2005 demonstrated a direct correlation between the blood vitamin D level and FEV_1_ and also between the blood vitamin D level and the forced vital capacity (FVC) [94]; this study has given impetus to focusing attention on the relationship of vitamin D deficiency to depressed respiratory function. A cross-sectional study conducted in 414 subjects published in 2010 demonstrated that decreased blood levels of vitamin D may have a bearing upon worsening of the airflow obstruction in COPD patients [86]. A study with highly precise measurement of the vitamin D concentration in blood samples from COPD patients (*n* = 433) and healthy subjects (*n* = 325) by liquid chromatography double mass spectrometry (LC-MS/MS) showed that vitamin D deficiency was more common among COPD patients than among healthy subjects, and that the FEV_1_ and blood levels of vitamin D were positively correlated in COPD patients [95]. Another cross-sectional study carried out in the general population (*n* = 2943) failed to reveal any significant relationship between the blood level of vitamin D and FEV_1_, probably attributable to the inferior statistical power of this study as compared with that of NHANES III, and to the low blood vitamin D levels in the entire study population [96]. In a 6-year longitudinal study performed to explore the relationship of the blood vitamin D level to the rate of progression of COPD, there was no significant difference in the baseline blood vitamin D level between COPD patients exhibiting a rapid decline of the FEV_1_ (rapid decliners) and those exhibiting a slow decline of the FEV_1_ (slow decliners) [97]. Blood vitamin D levels were corrected in this study for normal seasonal variations (the blood levels are elevated during summer on account of the higher exposure to sunlight, whereas they are decreased during winter because of the shorter exposure to sunshine during the winter months). It has been inferred that the limited hours of sunbathing in severe COPD patients may be responsible for the low blood vitamin D levels in these patients.

In 2012, the results of a RCT conducted on the ground of these observational studies to assess the preventive effect of vitamin D administration against exacerbation of COPD were reported [57]. High-dose vitamin D (100,000 IU) or placebo was administered to 186 patients with COPD at monthly intervals for a period of 1 year in this study. Blood vitamin D levels were elevated in the vitamin D-treated group, as compared to the levels in the placebo group; however, there was no significant intergroup difference with respect to the frequency of COPD exacerbation, FEV_1_, QOL or mortality rate between the groups. The results of a RCT of high-dose vitamin D treatment during respiratory rehabilitation in COPD patients have also been reported [98]. In this RCT, a more marked improvement of the inspiratory muscle strength and maximum oxygen intake was noted in the group of patients undergoing rehabilitation while receiving 100,000 IU of vitamin D at monthly intervals for 3 months, as compared to the placebo group. However, a retrospective, observational cohort study reported a reverse J-shaped relation between the serum level of 25(OH)D and all-cause mortality, with the lowest mortality risk at 50–60 nmol/L, suggesting that excessive vitamin D administration may have untoward effects [99].

The relation between genetic polymorphism of the vitamin D-binding protein (VDBP) with COPD has also been investigated [100,101]. For example, results of analysis of the genetic polymorphism of VDBP in patients with COPD demonstrated that the GC2 variant has a bearing on macrophage hypofunction, and that the rs7041T allele may be related to decline of the blood vitamin D level [102].

To sum up the above study results, COPD patients are frequently deficient in vitamins C, D and E, however, there is no consensus yet on whether the development or worsening of COPD might be prevented by administration of these vitamins.

## 9. Creatine Intake

Creatine is a nutrient that is abundantly contained in meats and fishes, but is often taken in the form of a dietary supplement, because it is decomposed on heating [103]. It is known that the muscle mass and muscle strength increase in healthy subjects receiving creatine concurrently with physical exercise training. In view of this, a number of RCTs of creatine supplementation combined with respiratory rehabilitation have been conducted in COPD patients [58,59,60]. Meta-analyses of these RCTs have, however, failed to show any add-on effect of creatine supplementation in improving the muscle strength of the upper and lower extremities, exercise tolerance (shuttle walk test and 6MWD) or HRQoL (SGRQ score) in COPD patients undergoing respiratory rehabilitation [61].

## 10. Intake of Other Nutrients

Diminished expression of nuclear factor erythroid 2-related factor 2 (Nrf2), a factor inducing antioxidant enzymes, has been implicated as a cause of augmented oxidative stress in COPD patients [104]. Sulforaphane, contained in such plants as broccoli and Japanese horseradish (*wasabi*), is known as a nutrient that fortifies Nrf2 [105]. It is also known that augmented oxidative stress leads to a decrease in histone deacetylase-2 (HDAC2) activity, with consequent enhancement of histone acetylation and eventual enhancement of inflammatory reactions and depressed HDAC2 activity, which in turn suppress the anti-inflammatory effects of corticosteroids in COPD patients. An *in vitro* study demonstrated that the yellow pigment curcumin in turmeric prevented monocytic HDAC2 expression and decreased the activity of HDAC2 caused by cigarette smoke extract [106]. Curcumin has been demonstrated to have the effect of augmenting the antioxidant capacity by stimulating GSH production [107].

Docosahexaenoic acid (DHA), which occurs in abundance in salmon and tuna fish, is an unsaturated omega-3 fatty acid, and patients with high blood DHA levels have been reported to show a reduced risk of development of COPD [108]. It has also been reported that peak exercise capacity and submaximal endurance time were increased after an 8 week rehabilitation program in COPD patients receiving a daily dose of 9 g polyunsaturated fatty acids (PUFA) compared to COPD patients receiving placebo [109]. A cohort survey of 250 COPD patients revealed that the blood TNF-α level was lower in a group of patients with high intake of omega-3 fatty acids, while the blood IL-6 and CRP levels were higher in the high omega-6 fatty acid intake group [110]. According to a cohort survey of 13,000 subjects in the Netherlands, however, no FEV_1_-maintaining effect of high omega-3 fatty acid intake was noted, whilst increased intake of omega-6 fatty acids was associated with a decrease of the FEV_1_ in tobacco smokers [111].

The effects on COPD patients of active intake of branched-chain amino acids (BCAA), which promote protein anabolism and are contained in rich abundance in milk and soybeans, have been examined. A clinical trial in COPD patients showed that intake of BCAA fortified to soy protein resulted in a significantly higher increase in whole-body protein synthesis than soy protein alone [112]. The results of a RCT showed increases in the body weight and FFM, decrease in the blood lactic acid level, and elevation of the arterial blood oxygen partial pressure in COPD patients receiving essential amino acids for three months [113]. Synthesis of muscle protein was shown to be diminished due to the deficiency of glutamine in subjects with enhanced protein catabolism [114], and COPD patients are known to be deficient in glutamate, a source of glutamine synthesis [115]. In a RCT of glutamine administration (0.125 g/kg bm) in COPD patients, however, no significant ameliorative effect of glutamine on the maximum oxygen uptake or lactate threshold on exercise was noted [62]. In a RCT conducted in 40 patients under mechanical ventilator due to exacerbation of COPD, the blood levels of IL-6, IL-8 and TNF-α did not differ between patients receiving daily administration of a glutamine solution for 5 days and those receiving administration of a placebo for the same period [63].

l-Carnitine, an amino acid derivative, promotes lipid combustion through its effect of mediating fatty acid transport into the mitochondria, consequently effecting increased energy production. In a RCT of 6-week respiratory rehabilitation with concurrent administration of l-carnitine (2 g/day, p.o.) or placebo in 16 COPD patients [64], greater degrees of improvement of the inspiratory muscle strength, 6 MWD and blood lactate level were noted in the l-carnitine-treated group. The study population, nevertheless, was small and further study is needed to confirm the ameliorative effect of l-carnitine on the exercise tolerance in COPD patients.

## 11. Conclusions

Undernutrition in patients with COPD is an important risk factor for deteriorated QOL and physical exercise performance, risk of exacerbation, and vital prognosis. The important causes of undernutrition in these patients include decreased dietary intake and increased energy expenditure associated with aggravation of the disease state of COPD, and also the effects of humoral factors such as inflammatory cytokines, adipokines, and hormones. Increasing attention has been focused particularly on the influence of adipokines on the systemic inflammation in COPD, besides the effects of adipokines in regulating the appetite and nutritional status. With the view to improving undernutrition in COPD patients, attempts are being made to encourage the intake of high-calorie foodstuffs, fruits and vegetables and intake of nutrients such as vitamins, amino acids and unsaturated fatty acids. It has been reported that these nutritional supplement therapies may not only improve undernutrition in COPD patients, but also provide various other beneficial effects such as prevention of the development, progression and exacerbation of COPD, and suppression of inflammation (Table 2). However, many previous studies have yielded inconsistent results, the reasons for which can be explained in several ways. First, the different results may be due to differences in the sample size, study design (*i.e.*, prospective or retrospective, cohort or intervention study) and duration of the studies (*i.e.*, short or long term). Second, the efficacy of nutritional supplement therapy in COPD patients may differ according to the differences in the severity and phenotype (*i.e.*, emphysematous or bronchitis phenotype) of the disease. Third, nutritional supplement therapy may not be equally beneficial in different COPD patients with different baseline nutritional levels; nutritional supplement therapy may be more efficacious in patients who are more deficient in the nutrient to be supplemented. Fourth, the effect of nutrition therapy for COPD patients may be confounded by supplements also providing other nutrients that may be beneficial in COPD. Nutritional supplement therapies whose efficacy has been verified by large-scale RCT and meta-analyses are still few, and further studies are needed. More efforts are also needed to explore new candidates (e.g., resveratrol and dietary fiber) for future trials of nutritional supplement therapies.
